# Genetic Variability and Selection Criteria in Rice Mutant Lines as Revealed by Quantitative Traits

**DOI:** 10.1155/2014/190531

**Published:** 2014-11-05

**Authors:** Yusuff Oladosu, M. Y. Rafii, Norhani Abdullah, Mohammad Abdul Malek, H. A. Rahim, Ghazali Hussin, Mohammad Abdul Latif, Isiaka Kareem

**Affiliations:** ^1^Institute of Tropical Agriculture, Universiti Putra Malaysia (UPM), Serdang, 43400 Selangor, Malaysia; ^2^Department of Crop Science, Faculty of Agriculture, Universiti Putra Malaysia (UPM), Serdang, 43400 Selangor, Malaysia; ^3^Department of Biochemistry, Faculty of Biotechnology and Biomolecular Science, Universiti Putra Malaysia (UPM), Serdang, 43400 Selangor, Malaysia; ^4^Bangladesh Institute of Nuclear Agriculture, Mymensingh 2202, Bangladesh; ^5^Bioscience and Agrotechnology Division, Malaysian Nuclear Agency, Bangi, 43000 Selangor, Malaysia; ^6^Strategic Livestock Research Centre, Malaysian Agricultural Research and Development Institute (MARDI), Serdang, 43400 Selangor, Malaysia; ^7^Bangladesh Rice Research Institute (BRRI), Gazipur, Bangladesh

## Abstract

Genetic based knowledge of different vegetative and yield traits play a major role in varietal improvement of rice. Genetic variation gives room for recombinants which are essential for the development of a new variety in any crop. Based on this background, this work was carried out to evaluate genetic diversity of derived mutant lines and establish relationships between their yield and yield components using multivariate analysis. To achieve this objective, two field trials were carried out on 45 mutant rice genotypes to evaluate their growth and yield traits. Data were taken on vegetative traits and yield and its components, while genotypic and phenotypic coefficients, variance components, expected genetic advance, and heritability were calculated. All the genotypes showed variations for vegetative traits and yield and its components. Also, there was positive relationship between the quantitative traits and the final yield with the exception of number of tillers. Finally, the evaluated genotypes were grouped into five major clusters based on the assessed traits with the aid of UPGMA dendrogram. So hybridization of group I with group V or group VI could be used to attain higher heterosis or vigour among the genotypes. Also, this evaluation could be useful in developing reliable selection indices for important agronomic traits in rice.

## 1. Introduction

Rice (*Oryza sativa*) is an important staple food in the world. Despite its position among the highly rated cereals, it feeds more than half of the world's population. The geometric growth rate of the global population has called for yield improvement of this very important cereal. On this, several methods have been tried by scientists to combat this perennial problem. Some researchers have tried nutritional method, physiological method, and breeding as well as control of pests and diseases. Among these methods, it is established that breeding for high yield traits is the most sustainable because the traits are heritable. To achieve this breeding objective, changes at cellular level are employed to get traits that could improve yield. These changes are referred to as mutations and may occur either in the genes or at chromosomal level to bring the desired improvement. At present, improvement of major food crops in the world rests majorly on mutation. This comes either naturally or through irradiation. So crops with restriction in genetic variation require mutagenesis or induced mutation to create desirable and heritable variations in them [1]. Following this, the use of induced mutations has been extensively used for genetic enhancement of different crops [2, 3]. Ionizing radiation of high frequencies and chemical mutagens are being employed to achieve genetic enhancement among various mutagenic agents available to induce favourable mutations. The mechanism of operation of ionizing radiation hinges on the level of the energy absorbed by the biological system of the plants or plant parts. The primary system which is the most important target in energy absorption is the chromosome [4]. Through this, there will be chromosomal changes which will manifest themselves in the subsequent progenies. The use of seed irradiation to achieve chromosomal changes has been found to increase frequencies of mutation and promote gene recombination which in turn promotes recombination and leads to mutation spectrum widening [5]. Based on radiation mutation, plant breeding has officially released over 2700 new crop cultivars in approximately 170 species [6].

Development of high-yielding cultivars requires a thorough knowledge of genetic variation in yield and its component among the existing cultivars. This is sine qua non to high-yielding cultivar development. Any variation observed in organisms is the result of combination of estimate of both genetic and environmental causes. Out of these two, only genetic cause is heritable. To get complete expectation of plant response to selection, only heritability estimate is not enough. So it should be combined with other estimates like genotypic and phenotypic coefficients of variation, genetic advance, and change in mean value between generations [7]. Extensive uses of gamma and x-rays in inducing mutations in crop plants have been well established. Therefore, successful adoption of this technology requires knowing the optimum dose for irradiating plant materials. At the same time, sound management of variations resulting from induction has to be embarked upon [8].

MR219 rice variety was developed by the Malaysian Agricultural Research and Development Institute (MARDI) and officially released in January 2001. It was the first variety to be developed by means of a direct seeding planting system. The emphasis was on the panicle characteristics. These are mainly the grain size and number of grains per panicle. As a result, a single grain of MR219 variety can weigh as much as 28–30 mg, and the number of grains can be as high as 200. This is higher than the previously released varieties. Other characteristics of this variety include short life cycle (105–111 days), fairly long but strong culms, and resistance to blast and bacterial leaf blight. Therefore, it could be marketed as a long-grain variety. In addition, cooked form of MR219 has soft texture (amylase content of 21.4%). This is the preference of most local consumers. It is the most cultivated variety grown in Malaysia, covering almost 90% of cultivated area [9].

Breeders are interested in evaluating genetic diversity based on morphological characteristics because they are inexpensive, rapid, and simple to score. The study of these characteristics does not require sophisticated equipment. Moreover, they can be inherited without specific biochemical or molecular techniques. Also, this evaluation could be useful in developing reliable selection indices for important agronomic traits in rice. Therefore, the present study was conducted to evaluate genetic diversity of derived mutant lines of MR219 and establish relationship between yield and yield components in the mutant lines using multivariate analysis.

## 2. Materials and Methods

### 2.1. Experimental Site and Plant Husbandry

Because MR219 is the most cultivated rice variety in Malaysia, covering almost 90% of the cultivated areas [9], efforts are constantly being made to increase its yield potentials. Therefore, its seeds were irradiated with an ion beam for radiosensitivity determination. A total of 100 seeds were subjected to 0, 10, 20, 40, 60, 80, 100, 120, 160, and 200 Gray (Gy) to determine the optimum doses for the production of high mutant frequency and spectrum. The optimum dose was found to be 60 Gy. The M_1_ seedlings were transplanted into the field with 25 cm × 25 cm planting distance. Ten thousand M_1_ seedlings were planted to produce M_2_ seeds and a total of 5,250 plants were selected from which 2 panicles per hill were randomly harvested from each hill. About 5% of M_2_ populations were selected for further screening in M_3_. After several series of selection and fixation, 31 potential lines with the required adaptive traits were recovered at M_4_ generation during the 2009–2012 seasons (M_0_–M_4_). In total, forty-five rice genotypes, which formed three populations (Malaysia, Vietnam, and Bangladesh), were analysed. Of these 45 genotypes, 31 were mutant lines derived from MR219, 4 were other commercial varieties from Malaysia, 8 were commercial mutant varieties from Bangladesh, and 2 were mutant varieties from Vietnam (Table 1). The field evaluation was carried out repeatedly in two locations on M4 generation. The first location was field 10 of the Universiti Putra Malaysia (UPM). It was 31 m above the sea level and is located between latitude 3°02′N and longitude 101°42′E. The second location was the farmers' field in Melor, Kelantan, Malaysia. It was located between latitude 5.9833°N and longitude 102.3°E. The climate of the two locations was a hot humid tropic. The areas fell under the rain forest. The periods of cultivation were December 2012 to April 2013 and February to June 2013. 30 g of each rice accession was placed in plastic petri dishes and oven-dried at 50°C for 24 hours to break seed dormancy. The seeds were then pregerminated and finally transferred into a nursery for proper establishment. 21-day-old seedlings were then transplanted into a field of size 22 m by 18 m. The plant spacing was 25 cm by 30 cm with density of two seedlings per stand. This experiment was laid out in randomized complete block (RCB) with three replications. The fields were irrigated throughout the experiment with average of 10 cm water above the soil level. Regular hand weeding was embarked upon to free the plant of interspecific competition. Fertilizer was applied in splits using 57 kg/ha triple super phosphate, 42 kg/ha muriate of potash, and 80 kg/ha urea at 15, 35, 55, and 75 days after planting. Insecticides (Malathion and Hopper Gun) were applied for controlling insect pests as required at recommended rates.

### 2.2. Data Collection

From each variety, five plants were randomly sampled from each block for data collection. Data collection was on plant height, flag leaf length to width ratio, number of tillers per hill, days to flowering, days to maturity, number of panicles per hill, panicle length, total number of grain per panicle, 100 grain weight, total grain weight per hill, seed length, seed length to width ratio and yield in t/ha (Table 2).

### 2.3. Variance Components

Variance components were estimated to determine genetic variation among genotypes and to assess genetic and environmental effects on different traits. The variance components were calculated as follows.

### 2.4. Genotypic Variance (*σ*_*g*_^2^)

Consider

(1) $$\begin{array}{c} \sigma_{g}^{2}=\frac{\left(\text{MSG}-\text{MSE}\right)}{r}, \end{array}$$

where MSG is mean square of genotypes, MSE is mean square of the error, and *r* is number of replications.

### 2.5. Phenotypic Variance (*σ*_*p*_^2^)

Consider

(2) $$\begin{array}{c} \sigma_{p}^{2}=\sigma_{g}^{2}+\sigma_{e}^{2}, \end{array}$$

where *σ*_*g*_^2^  is genotypic variance and *σ*_*e*_^2^ is the mean squares of error.

### 2.6. Error Variance (*σ*_*e*_^2^)

Consider

(3) $$\begin{array}{c} \sigma_{e}^{2}=\text{MSE}, \end{array}$$

where MSE is the mean square of error.

### 2.7. Phenotypic and Genotypic Coefficient of Variation (PCV and GCV)

The estimates of phenotypic and genotypic coefficient of variation were calculated according to Singh and Choudhary [10] as follows:

(4) $$\begin{array}{c} \text{PCV}=\frac{\sqrt{\sigma_{P}^{2}}}{\overset{-}{X}}\times 100,\\ \text{GCV}=\frac{\sqrt{\sigma_{g}^{2}}}{\overset{-}{X}}\times 100. \end{array}$$

*σ*_*P*_^2^ is the phenotypic variance. *σ*_*g*_^2^ is the genotypic variance. $\overset{-}{X}$ is the mean of the trait. GCV and PCV values were categorized as low (0–10%), moderate (10–20%), and high (20% and above) following Sivasubramanian and Madhava Menon [11].

### 2.8. Heritability Estimate

This heritability *h*_*B*_^2^ (broad sense) is the ratio of genetic variance (*σ*_*g*_^2^) to phenotypic variance (*σ*_*p*_^2^) [12]. It is calculated as

(5) $$\begin{array}{c} h_{B}^{2}=\frac{\sigma_{g}^{2}}{\sigma_{p}^{2}}, \end{array}$$

where *σ*_*g*_^2^  is the genotypic variance and *σ*_*p*_^2^ is the phenotypic variance.

The heritability percentage was categorized as low (0–30%), moderate (30–60%), and high (≥60%) in accordance with Robinson et al. [13].

### 2.9. Estimated and Expected Genetic Advance

Expected genetic advance (GA) (as percentage of the mean) was calculated using the method of Assefa et al. [14] and selection intensity (*K*) was assumed to be 5%. Genetic advance was categorized as low (0–10%), moderate (10–20%), and high (>20%) by following [15].

(6) $$\begin{array}{c} \text{GA}=K\times \sqrt{\sigma_{p}^{2}}\times h_{B}^{2},\\ \text{GA}\left(\%\right)=K\times \frac{\sqrt{\sigma_{P}^{2}}}{\overset{-}{X}}\times h_{B}^{2}\times 100. \end{array}$$

*K* is a constant which represents the selection intensity. When *k* is 5%, the value is 2.06. $\sqrt{\sigma_{P}^{2}}$ is phenotypic standard deviation, *h*_*B*_^2^ is the heritability, and $\overset{-}{X}$ is the mean of traits.

### 2.10. Cluster Analysis

In this study, cluster and principal component analysis (PCA) were used to assess the genetic diversity of quantitative traits [16]. Cluster analysis grouped individuals on the basis of their characteristics. So individuals having similar characteristics were mathematically clustered together using distance, similarity, and relatedness of varieties as the basis. On the basis of distance, clustering was done.

### 2.11. Data Analysis

All the morphological and yield data collected were subjected to analysis of variance (ANOVA) while significant means were separated with least significant difference (LSD) using SAS 9.1 software. Also mean, range, standard deviation, and coefficient of variation (CV) were recorded for each trait measured. The relationships among the traits were also determined using correlation analysis. Genetic variance data generated were analysed based on Euclidian distance method, Dice's and Jaccard's similarity coefficient. Genetic relationships among the rice genotypes were determined using UPGMA algorithm and SAHN methods. All these were achieved with NTSYS-pc 2.1 software.

## 3. Results

### 3.1. Genetic Variation for Vegetative Characters

From the pooled data of the two sites, there were significant differences among yield components. Five parameters related to plant vegetative growth were analysed for variation assessment (Table 3). The varietal impact on plant height was significant for all the genotypes (*P* < 0.01). The plant height varied from 147.67 to 71.74 cm. The tallest plant (147.67 cm) was from Binasail, whereas the shortest plant (71.74 cm) was from IRATOM-38. The genotypes MR 219, MR 220, ML-16, ML-11, ML-24, ML-7, ML-18, ML-19, ML-22, ML-25, ML-4, ML-27, ML-31, ML-30, ML-5, and ML-29 were similar in height. Their height was an intermediate one. The values for flag leaf length to width ratio (FLWR) ranged from 72.89 to 22.32. The Binasail genotype had the highest value, whereas VN121 had the lowest value. In the case of number of tillers (NT) per hill, the values were between 26 and 14. The highest number of tillers (26) was from Binasail whereas the lowest number (14) was from ML-7 and ML-12. However, ML-3, ML-16, ML-24, ML-17, ML-6, ML-9, ML-30, ML-15, and ML-28 were statistically equal to one another. The number of days to 50% flowering was between 53 and 77 days, as observed in IRATOM-38 and ML7, respectively. Days to maturity varied significantly (*P* < 0.01) among the genotypes and range was from 85 to 124 days. The earliest maturing (85 days) genotype was IRATOM-38 while genotype Binasail matured last (124 days). ML-2, ML-5, ML-17, ML-29, ML-31, ML-1, ML-2, ML-3, ML-16, ML-13, ML-18, ML-6, ML-21, ML-27, ML-20, MR 220, and ML-9 had similar average number of days to maturity.

### 3.2. Yield and Its Components

Eight parameters on rice yield were analysed for genetic variability (Table 3). The number of panicles per hill varied from 21 to 13. The highest number of panicles (21) was found in Binasail, which was statistically similar to Binadhan-5, Binadhan-8, IRATOM-38, VN121, VNI24, and Binadhan-10. The lowest number of panicles (13) was observed in ML12 (12.83) which was statistically similar to ML7. However, genotypes ML-29, ML-19, ML-22, ML-2, MR 219, MR 264, MR 253, Binadhan-6, Binadhan-7, ML-5, ML-30, ML-11, and ML-14 had similar number of panicles. The differences in the number of panicles produced among the genotypes were significantly significant (*P* < 0.01). The panicle length ranged from 31.33 to 22.33 cm and the highest (31.33 cm) was observed in Binasail followed by Binadhan-4 (26.67 cm) while the shortest panicle length (22.33 cm) was recorded in VN124 which was statistically similar to Binadhan-5, ML-14, ML-2, and IRATOM-38. The highest number of filled grains (181) was from ML-21 while the lowest (65) was from Binadhan-7. A significant difference was observed for grain per panicle among all varieties, with values that ranged from 220 to 91. The highest number of grain per panicle (220) was observed in Binasail, which was statistically similar to ML-10 and ML-4 but differed from the rest of the genotypes. The lowest number of grains per panicle (91) was observed in Binadhan-10. The highest amount of total grain weight per hill belonged to ML-9, whereas ML-12 had the lowest amount of total grain weight per hill. The 100 grain weight varied significantly among the genotypes, with the weight ranging from 2.85 to 1.73 g. ML-3 had the highest value of 100 grain weight (2.85 g), which was not significantly different from ML-10, ML-21, ML-9, MR 220, and Binadhan-8. The lowest 100 grain weight (1.73 g) was recorded in Binasail (Table 3). The seed length also varied significantly among the genotypes, ranging from 8.31 to 5.11 mm. ML-30 had the longest (8.31 mm) seeds, whereas Binasail had the shortest (5.11 mm) seeds. The grain shape varied significantly among the genotypes, with values ranging from 2.4 to 1.66 mm. The highest seed length to width ratio (2.4 mm) was found in ML-14, whereas the lowest value (1.66 mm) was observed in Binasail. The highest yield/ha (7.12 ton/ha) was found in genotype ML-9, followed by ML-4 (6.72 ton/ha), whereas the lowest value (2.87 ton/ha) was observed in ML-12 (Table 3).

### 3.3. Heritability, Selection Gain, and Coefficient of Variation

In this study, broad sense heritability was high for all the traits except total grain weight (28%), yield per hectare (44%), and seed length to width ratio (45%) (Table 4). The highest heritability value (98%) was found in 100 grain weight, whereas days to flowering, days to maturity, and plant height had 91%, 91%, and 89% values, respectively. In addition, high level of variability was observed in genotypic coefficient of variation (GCV) and phenotypic coefficient of variation (PCV). The high values of GCV were recorded from flag leaf length to width ratio (28.31%) and yield per hectare (22.53%) whereas the low values were found in panicle length (7.2%), 100 grain weight (9.49%), and days to maturity (9.66%). The highest PCV value was found in yield per hectare (34.14%) whereas the lowest value was recorded from panicle length (8.78%). Furthermore, the genetic advance (GA) for 13 traits had its peak with the flag leaf length to width ratio (55.77%) followed by the plant height (38.57%), whereas the lowest value were recorded from the panicle length (12.15%) and from seed length to width ratio (14.18%) (Table 4).

### 3.4. Relationship between Traits

Correlation coefficients among morphological traits and yield and its components showed that all the traits had a positive correlation with yield, except for the number of tillers (Table 5). The total grain weight significantly correlated with the flag leaf length to width ratio, days to flowering, days to maturity, leaf area, and panicle length (Table 5).

### 3.5. Cluster Analysis of Morphological Traits

**Figure 1:**
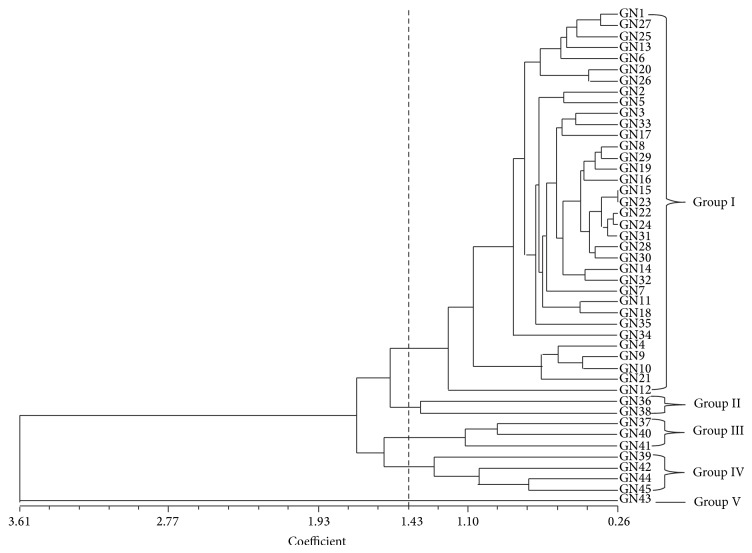
Clustering pattern of the morphological trait at dissimilarity coefficient of 1.43.

The standardized morphological data were employed to calculate the Euclidean distances among the 45 genotypes and a UPGMA dendrogram was constructed using these values as indicated in Table 6 and Figure 1. In this dendrogram, the 45 rice genotypes were grouped into 5 primary clusters based on 13 morphological traits. Among the five clusters, cluster I had the largest number of genotypes (35), cluster II had 2 genotypes, and cluster III had 3 genotypes. Cluster IV had 4 genotypes whereas cluster V contained only a genotype.

## 4. Discussion

The traits assessed in 45 rice accessions showed significant differences among themselves. This pointed at the existence of variation in the population. The differences exhibited by the genotypes could be because they originated from different areas. In this direction, several reports have been published on phenotypic variation among rice genotypes [17–20]. Similarly, Pandey et al. [21] reported highly significant differences among 40 rice accessions with the use of 12 quantitative characters. In the same vein, [22] discovered 95% differences among five rice populations by using 20 morphological characters. The high-yielding genotypes were short. This feature was as a result of short internode. This could equally be attributed to very effective assimilate partitioning at the expense of vegetative growth. So, instead of having tall plants, high yield came as a compensation for the vegetative deficiency. This trait is also advantageous in protection against lodging. Though plant height is mostly governed by the genetic makeup of the genotype, it is highly influenced by environmental factors. As indirectly pointed out earlier, rice yield is indirectly related to its height. This is due to sink competition for the limited photosynthates produced by limited sources. So what will be used for yield increase will be unnecessarily used for somatic cell enlargement that results in luxuriant vegetative growth and enhanced height. Therefore, tall varieties normally have lower yield than the short ones. Another serious disadvantage of tallness rice is lodging which significantly lowers the final yield and makes the plants prone to some other natural attacks. In this experiment, all the high-yielding varieties were found to be of intermediate height. This implies that moderate plant height is desirable when breeding for high-yielding varieties. Flag leaf plays a significant role in enhancing rice yield because it remains the only source of assimilate production for the filling spikelets during grain-filling stage [23, 24]. The larger the leaf area, the more the solar interception and photosynthate production provided that all other factors of production are not limiting. Therefore, flag leaf area was found to be directly related to the yield components: number of panicles, panicle length, number of grains per panicle, 100 grain weight, total grain weight per hill, and yield per hectare. Furthermore, the flag leaf has been found to be metabolically active to support higher grain yield. Corroborating our finding in this work, Ashrafuzzaman et al. [25] have made it clear that yield components positively correlate with flag leaf area. Number of tillers plays a significant role in determining yield of the rice grain since it is directly related to panicle number that will be produced per unit ground area. Fewer tillers result in fewer panicles; excess tillers cause high tiller abortions, small panicles, poor grain filling, and reduction in grain yield [21]. It has been observed that leaf area index and plant nitrogen status are the two major factors that affect tiller production in rice crops [21]. When there is adequate nutrient supply, mitotic cell division will be enhanced and growth of tillers and plant general vegetative life will receive a boost. In this work, the tiller production was between moderate and low levels. So the case of tiller abortion was not a problem during production period. The number of panicles per hill was between moderate (21) and low (13). This correlates with the number of tillers produced. So the number of effective tillers rests on the number of tillers produced and this is directly proportional to the panicles produced per unit area and finally depends on variety [26]. The panicle number is an important character which directly influences the yield. Therefore, yield could be increased when agronomic manipulation is used to increase the number of panicles produced per unit area. Among yield contributing characters is panicle length. This is proportional to the number of potential spikelets to be filled during grain-filling stage.

Rice plants will naturally have the superior spikelets filled before the inferior ones. The superior spikelets are located on the primary branches located at the apex of the rice plant. So they fill faster and produce heavier and larger grain weight. On the other hand, inferior spikelets are usually located on secondary branches and are usually slow in filling. They also produce poorly filled spikelets or sterile ones [27]. Slow grain-filling rate and low grain weight of inferior spikelets have often been attributed to limitation in carbohydrate supply [27]. According to [28], the fundamental factors responsible for variations in grain filling between the superior and inferior spikelets remain unknown. As it could be seen from this study, some varieties flower earlier than the others. Those that flowered earlier matured early while those that flowered late had a delay in their maturity. Early flowering indicates short life cycle and is considered a positive character for rice improvement. Early maturing varieties are advantageous in areas with short rainfall duration because they grow faster during the vegetative phase and are thus more competitive with weeds. They reduce weed control costs and utilize less water [29]. It is a known fact that drought frequently impedes production of rain-fed lowland rice [30]. So when drought occurs towards the reproductive stage of rice production, pollination, and fertilization as well as grain filling are severely affected and panicle blanking may result. In the situation, early maturing variety will give remedial measures in lieu of establishment of irrigation facilities and development of drought-tolerant varieties [30].

The number of filled grains among the varieties ranged from high (181) to low (65). Increase in number of filled grains could be attributed to efficient translocation of carbohydrates from the sources to the spikelets (sinks) which consequently leads to increase in grain yield [31]. The varietal yield in this work was between high and low. Yield differences among different rice varieties have been reported anytime a comparison is made between different varieties of rice in both field and glasshouse trials [32]. The differences are genetically based, though environment has a great contribution in the manifestation of the inherent potential. In this work, the genotypes with higher number of effective tillers as well as higher number of grains per panicle also had higher yield. This is in line with the observation of [33]. The weight of 100 or 1000 grain weight contributes significantly to the final yield per unit area. It represents the weight of individual seeds which could not be directly measured because of the size of individual seeds. The result of the present study showed that 100 grain weight varied significantly among the tested varieties. This could also be due to their differences in origin and genetic makeup. Similar reports have been published by a BRRI scientist [34] as well as Ashrafuzzaman et al. [25]. Panicle length determines how many spikelets will be found in a panicle and therefore filled spikelets and consequently final grain yield. The longer the panicle, the more the spikelets and the filled grains, if other environmental conditions are not limiting. As found here, panicle length correlated positively with the final yield. Credence to this research finding has been laid by [35] who also found a significant positive association between panicle length and grain yield per hill.

Heritability is the proportion of phenotypic traits (physical appearance) or total variance that is inherited from the parents. Higher genotypic coefficient of variation together with high heritability as well as high genetic advance gives better clues than individual parameters. Thus, the traits with high genotypic coefficient of variation, heritability, and genetic advance are selected. In this study, flag leaf length to width ratio, plant height, and the total number of grains per panicle had higher values for genotypic coefficient of variation, heritability, and genetic advance. Therefore, selection with a view to develop one trait which will positively influence other traits is of paramount importance.

Having positive correlation coefficients between the vegetative traits and the yield parameters in this work was an indication that the quantitative traits measured were appropriate for yield prediction and selection for breeding programmes to obtain better vigour or heterosis. The positive correlation between the final yield and plant height and total number of grain per panicle and days to maturity indicates that better exploration of these three traits could be used to develop desired genotypes.

The contribution of individual panicle grain yield sums up to produce the final yield. Therefore, high panicle grain yield could be successfully used as an important selection index for grain yield [36]. Furthermore, when the panicle yield is correlated with the yield per unit area, positive correlation coefficient will result [37]. In the present study, yield per panicle had the best relationship with yield production. Hence, more attention is given to this trait for final yield determination in all the assessed genotypes.

The UPGMA dendrogram broadly clustered the rice genotypes into five major groups at 1.43 dissimilarity coefficients. This implies a high level of morphological diversity in the rice genotypes. This study revealed effectiveness of quantitative or morphological traits in grouping rice genotypes. It has been established that genetic divergence analysis among rice genotypes on the basis of morphological traits can be used to classify and differentiate different genotypes in a population [38]. This genetic divergence analysis also plays a vital role in selection of diverse genotypes for the further improvement of rice varieties through breeding [39]. We have succeeded in clustering 45 genotypes into 5 major groups using some quantitative traits for better handling of the diverse genotype as well as better selection for acquisition of higher heterotic vigour in the resulting offspring after crossing. Similarly, Ahmadikhah et al. [40] clustered 58 rice varieties into four groups using 18 morphological traits, while Mazid et al. [41] had his 41 rice genotypes clustered into 6 on the basis of 13 morphological traits. Therefore, to attain success in breeding for higher vigour among the genotypes, group I could be hybridized with group V or group VI in this work and as corroborated by findings of [41, 42].

As observed in this study, the experimented rice genotypes showed notable genetic diversity in terms of morphological traits. This shows the efficacy of ion beam radiation in inducing high performing agronomic traits in some mutant lines as compared to the control. High heritability together with high genetic advance was obtained for plant height, flag leaf length to width ratio, days to maturity, and total numbers of grains per panicle.

## 5. Conclusion

The development of rice genotypes that could lead to attainment of food security rests on exploring genetic aspects of its quantitative traits. This is because genetic variations give room for recombinants which are essential in development of new genotype. Field trials were carried out on 45 mutant rice genotypes and their genetic diversities were determined using their quantitative traits. All the genotypes showed variations for vegetative and yield traits. Also, all the traits related positively with the final yield with the exception of number of tillers. Finally, the evaluated genotypes were grouped into five major clusters based on the assessed traits with the aid of UPGMA dendrogram. So hybridization of group I with group V or group VI could be used to attain higher heterosis or vigour among the genotypes. This evaluation could be useful in developing reliable selection indices for important agronomic traits in rice. It is recommended that future research explore molecular means to further confirm the outcome of this research and establish the relationship between the two methods.

## Figures and Tables

**Table 1:** List of rice genotypes studied.

Genotype code	Name of accession	State	Mutagenesis source
GN1	ML-1	Mutant line	Ion beam
GN2	ML-2	Mutant line	Ion beam
GN3	ML-3	Mutant line	Ion beam
GN4	ML-4	Mutant line	Ion beam
GN5	ML-5	Mutant line	Ion beam
GN6	ML-6	Mutant line	Ion beam
GN7	ML-7	Mutant line	Ion beam
GN8	ML-8	Mutant line	Ion beam
GN9	ML-9	Mutant line	Ion beam
GN10	ML-10	Mutant line	Ion beam
GN11	ML-11	Mutant line	Ion beam
GN12	ML-12	Mutant line	Ion beam
GN13	ML-13	Mutant line	Ion beam
GN14	ML-14	Mutant line	Ion beam
GN15	ML-15	Mutant line	Ion beam
GN16	ML-16	Mutant line	Ion beam
GN17	ML-17	Mutant line	Ion beam
GN18	ML-18	Mutant line	Ion beam
GN19	ML-19	Mutant line	Ion beam
GN20	ML-20	Mutant line	Ion beam
GN21	ML-21	Mutant line	Ion beam
GN22	ML-22	Mutant line	Ion beam
GN23	ML-23	Mutant line	Ion beam
GN24	ML-24	Mutant line	Ion beam
GN25	ML-25	Mutant line	Ion beam
GN26	ML-26	Mutant line	Ion beam
GN27	ML-27	Mutant line	Ion beam
GN28	ML-28	Mutant line	Ion beam
GN29	ML-29	Mutant line	Ion beam
GN30	ML-30	Mutant line	Ion beam
GN31	ML-31	Mutant line	Ion beam
GN32	MR 219	Released variety	Conventional method
GN33	MR 220	Released variety	Conventional method
GN34	MR 253	Released variety	Conventional method
GN35	MR 264	Released variety	Conventional method
GN36	Binadhan-4	Released variety	Gamma ray
GN37	Binadhan-5	Released variety	Gamma ray
GN38	Binadhan-6	Released variety	Gamma ray
GN39	Binadhan-7	Released variety	Gamma ray
GN40	Binadhan-10	Released variety	Gamma ray
GN41	Binadhan-8	Released variety	Gamma ray
GN42	IRATOM-38	Released variety	Gamma ray
GN43	Binasail	Released variety	Gamma ray
GN44	VN-124	Released variety	Gamma ray
GN45	VN-121	Released variety	Gamma ray

NB: ML: mutant line.

**Table 2:** List of quantitative traits.

Growth trait/yield trait	Denotation	Method of evaluation
Plant height	PH (cm)	The average height from the base to the tip of last leaf (flag leaf)
Flag leaf length to width ratio	FLWR (cm)	Divide the flag leaf length by width
Number of tillers per hill	NT (number)	Count the number of tillers per hill
Days to flowering	DF (days)	Count the number of days from seeding to flowering
Days to maturity	DM (days)	Count the number of days from seeding to maturity
Number of panicles per hill	NP/H (number)	Count the number of panicles per hill
Panicle length	PL (cm)	Measure the length from the node below the lowest branch on the panicle to the top of first superior spikelet
Total number of grain per panicle	TNG/P (number)	Count the number of spikelets per panicle
Hundred grain weight	100 GW (g)	Weigh any 100 filled grains
Total grain weight per hill	TGW/H (g)	Weigh total grains produced per hill
Seed length	SL (cm)	Measure the length of the seeds using microscopic picture and Leica application suit software
Seed length to width ratio	SLWR (cm)	Divide the seed length by width
Yield in t/ha	Y/ha (ton)	Number of tillers per square meter × average total grains per tillers divided by 100

**Table 3:** Morphological traits, yield and yield components of rice accession for the two location.

Accession	PH, (cm)	FLR, (cm)	NT (no.)	DF, (days)	DM, (days)	NP/H, (no.)	PL, (cm)	TNG/P, (no.)	100 GW, (g)	TGW/H (g)	SL, (mm)	SLWR (mm)	Y/ha, (t/ha)
ML-1	88.80	34.17	19.5	72.5	111.5	17.8	24.17	141.2	2.55	33.26	7.52	2.22	5.32
ML-2	86.13	39.27	15.8	72.0	111.0	15.7	22.83	145.7	2.31	26.60	7.18	2.13	4.26
ML-3	89.07	40.32	17.8	72.8	111.8	17.0	24.17	157.0	2.85	30.22	8.06	2.34	4.84
ML-4	84.27	47.37	14.5	71.8	106.7	14.0	23.17	204.7	2.67	42.21	7.65	2.29	6.72
ML-5	83.47	38.86	16.2	72.2	111.2	15.5	24.00	142.5	2.47	21.12	7.76	2.25	3.38
ML-6	91.27	41.09	17.3	73.7	112.7	16.2	24.33	152.3	2.41	40.48	7.95	2.32	6.48
ML-7	85.27	39.65	14.0	76.5	115.5	13.2	24.67	157.7	2.42	28.80	7.68	2.26	4.61
ML-8	82.73	40.44	18.5	75.3	114.3	16.2	24.67	156.3	2.52	32.27	7.63	2.27	5.16
ML-9	88.87	45.89	17.3	75.7	113.3	16.2	24.50	187.8	2.72	44.55	7.77	2.25	7.13
ML-10	90.40	48.26	15.8	73.3	110.3	16.2	23.83	206.8	2.81	40.79	7.60	2.30	6.53
ML-11	86.53	38.79	15.2	75.3	114.3	15.3	24.17	152.5	2.48	29.46	7.32	2.14	4.71
ML-12	87.07	33.98	14.0	75.8	114.8	12.8	23.50	130.2	2.51	17.91	7.29	2.15	2.87
ML-13	88.67	35.54	18.3	73.3	112.3	16.5	23.83	160.0	2.63	39.65	7.62	2.19	6.34
ML-14	89.27	45.84	16.7	70.0	109.0	15.0	22.83	141.2	2.42	29.47	7.86	2.40	4.72
ML-15	88.00	42.22	17.2	71.8	110.8	16.5	24.33	157.3	2.51	26.81	7.45	2.20	4.29
ML-16	86.73	35.37	17.8	73.3	112.3	17.2	25.33	172.3	2.54	32.91	7.78	2.32	5.27
ML-17	84.87	43.95	17.3	72.2	111.2	14.8	24.17	160.0	2.55	28.71	7.76	2.27	4.59
ML-18	85.27	36.73	15.2	73.5	112.5	14.5	25.00	163.2	2.55	31.68	7.88	2.36	5.07
ML-19	84.40	40.82	16.3	73.0	110.3	15.8	24.33	142.7	2.44	33.72	7.67	2.30	5.40
ML-20	87.73	39.97	19.7	73.5	113.2	19.7	24.67	146.5	2.62	27.97	8.11	2.34	4.48
ML-21	80.27	44.15	18.8	75.5	112.8	17.8	24.67	199.2	2.75	39.79	7.88	2.34	6.37
ML-22	84.33	38.66	16.5	74.8	113.8	15.8	24.50	160.8	2.46	27.32	7.82	2.18	4.37
ML-23	87.60	42.31	16.7	73.2	112.2	16.7	24.67	166.0	2.41	29.50	7.61	2.21	4.72
ML-24	86.13	36.46	17.7	74.8	113.8	17.0	24.17	158.8	2.42	28.54	7.58	2.24	4.57
ML-25	84.27	40.54	18.0	76.0	115.0	18.7	24.17	163.5	2.63	38.48	7.57	2.25	6.16
ML-26	89.87	34.95	19.3	76.5	115.5	19.5	24.50	158.5	2.51	32.07	8.01	2.34	5.13
ML-27	84.13	37.62	18.8	74.0	113.0	18.3	24.50	153.0	2.48	36.83	7.73	2.26	5.89
ML-28	91.80	41.72	17.2	75.0	114.0	16.8	24.67	152.8	2.50	29.50	7.99	2.40	4.72
ML-29	83.33	39.37	18.3	72.2	111.2	15.8	24.67	155.2	2.59	31.73	7.95	2.35	5.08
ML-30	83.47	39.50	17.3	75.3	114.3	15.3	24.67	155.8	2.42	27.34	8.31	2.36	4.38
ML-31	83.67	36.13	16.8	72.3	111.3	16.3	24.50	167.8	2.43	29.23	8.00	2.31	4.68
MR 219	85.27	40.39	15.7	76.0	115.0	15.7	23.33	143.8	2.38	30.52	7.85	2.34	4.88
MR 220	85.87	41.08	16.8	74.3	113.3	14.7	24.67	142.5	2.71	28.74	7.87	2.33	4.60
MR 253	82.60	29.29	16.8	67.2	106.2	15.5	24.67	129.3	2.66	26.62	7.85	2.22	4.26
MR 264	81.40	36.67	15.8	71.7	110.7	15.7	24.50	111.3	2.46	34.84	7.91	2.32	5.58
Binadhan-4	128.50	52.54	19.8	64.8	95.8	17.2	26.67	148.7	2.63	34.27	7.52	2.06	5.48
Binadhan-5	99.47	43.90	19.8	59.2	98.2	18.3	22.83	102.8	2.40	31.18	6.65	1.98	4.99
Binadhan-6	110.50	42.76	14.2	62.5	101.5	15.5	25.50	139.2	2.27	34.83	5.87	1.73	5.57
Binadhan-7	73.20	27.63	16.0	55.5	94.5	15.5	23.67	122.7	2.24	20.62	6.93	1.98	3.30
Binadhan-10	81.80	34.08	21.0	61.2	100.2	19.7	23.17	91.2	2.54	30.16	6.39	1.84	4.83
Binadhan-8	96.93	54.31	18.3	55.3	94.3	19.5	23.67	99.7	2.81	28.38	5.68	1.71	4.54
IRATOM-38	71.47	31.41	22.0	53.2	85.8	18.8	22.67	127.5	2.48	21.33	6.45	1.83	3.41
Binasail	147.70	72.89	26.2	64.2	124.7	21.7	31.33	220.3	1.73	24.84	5.12	1.67	3.98
VN-124	78.40	22.98	22.5	57.7	96.7	21.3	22.33	111.2	2.39	21.38	7.65	2.26	3.42
VN-121	79.00	22.32	19.3	56.2	95.2	19.5	23.50	135.5	2.27	29.29	7.36	2.14	4.69
LSD (*P* ≤ 0.05)	6.85	3.92	2.1	3.15	3.6	2.8	1.40	25.7	0.14	9.18	0.44	0.28	1.47

**Table 4:** Mean squares of morphological traits, yield and yield component of the rice accession.

S.O.V.	Df	PH, cm	FLWR, cm	NT, (no.)	DF, days	DM, days	NP/H, (no.)	PL, (cm)	TNG/P, (no.)	TGW/H, (g)	100 GW, (g)	SL, (mm)	SLWR, (mm)	Y/ha, (t/ha)
Reps/Site (R/S)	4	968.43^**^	581.02^**^	32.90^**^	84.46^**^	80.92^**^	51.2^**^	3.5	2803.7	67.2	0.01^**^	0.42	0.23	1
Genotypes (G)	44	955.61^**^	393.16^**^	32.95^**^	265.79^**^	344.21^**^	23.2^**^	10.7^**^	2889.9^**^	154.9	0.17^**^	2.64^**^	0.21^**^	5.3^**^
Sites (S)	1	43.2	413.23^**^	216.90^**^	482.67^**^	229.63^**^	604.5^**^	580.8^**^	125539.5^**^	290.1	0.001	0.001	31.67^**^	2.5
G × S	44	0.01	6.19	16.35^**^	52.38^**^	30.37^**^	14.2^**^	2.9	867.4	0.3	0.001	0.001	0.82^**^	0.4
Error	176	36.17	11.84	3.34	7.66	9.87	5.8	1.5	459.4	70.6	0.001	0.15	0.06	1.6

Mean		88.44	39.83	17.65	70	109	16.73	24.33	151	30.79	2.5	7.49	2.19	4.93
GV		306.48	127.11	9.87	86.04	111.45	5.8	3.07	810.17	28.1	0.06	0.83	0.05	1.23
PV		342.65	138.95	13.21	93.7	121.32	11.6	4.57	1269.57	98.7	0.06	0.98	0.11	2.83
PCV (%)		20.93 (h)	29.59 (h)	20.59 (h)	13.76 (m)	10.08 (m)	20.36 (h)	8.78 (l)	23.6 (h)	32.27 (h)	9.58 (l)	13.22 (m)	15.14 (m)	34.14 (h)
GCV (%)		19.79 (m)	28.31 (h)	17.8 (m)	13.18 (m)	9.66 (l)	14.4 (m)	7.2 (l)	18.85 (m)	17.22 (m)	9.49 (l)	12.16 (m)	10.21 (m)	22.53 (h)
*h*_*B*_^2^ (%)		89 (h)	91 (h)	75 (h)	92 (h)	92 (h)	50 (m)	67 (h)	64 (h)	28 (l)	98 (h)	85 (h)	45 (m)	44 (m)
GA (%)		38.57	55.77	31.69	26.02	19.07	20.97	12.15	31.02	18.92	19.39	23.06	14.18	30.62

^*^Significant at 0.05 probability level, ^**^highly significant at 0.01 probability level, S.O.V: source of variation, df: degree of freedom, PH: plant height; FLR: flag leaf length to width ratio; NT: number of tillers per hill; DF: days to flowering; DM: days to maturity; NP/H: number of panicles per hill; PL: panicle length; NPB/P: number of primary braches per panicle; TNG/P: total number of grain per panicle; NUFG/P: number of unfilled grain per panicle; TGW/H: total grain weight per hill; 100 GW: one hundred grain weight; SL: seed length; SLWR: seed length to width ratio; Y/ha: yield in t/ha. GV: genotypic variance, PV: phenotypic variance, PCV: phenotypic coefficient of variation, GCV: genotypic coefficient of variation, *h*_*B*_^2^: broad sense heritability, GA: genetic advance (as percentage of mean), ^*^significant at 0.05 level, and ^**^highly significant at 0.01 level. (h: high, m: moderate, l: low).

**Table 5:** Correlation coefficient among 16 quantitative traits for 45 rice accessions.

	FLR	NT	DF	DM	NP	PL	TNG	GW	TGW	SL	SLWR	YTHA
PH	0.62741^**^	0.28782^**^	−0.1204^*^	0.0958	0.16758^*^	0.41755^**^	0.1498^*^	−0.35534^**^	0.06722	−0.42857^**^	−0.13489^*^	0.06656
FLR		0.13341^*^	0.06756	0.2601^**^	0.06049	0.41032^**^	0.31058^**^	−0.12583^*^	0.18743^*^	−0.29327^**^	−0.12007^*^	0.1891^*^
NT			−0.29697^**^	−0.13746^*^	0.74554^**^	0.25826^**^	0.11484	−0.19125^*^	−0.00905	−0.2171^**^	−0.30109^**^	−0.00893
DF				0.85622^**^	−0.24661^**^	−0.05954	0.25831^**^	0.20724^**^	0.19197^*^	0.50885^**^	0.17302^*^	0.19181^*^
DM					−0.13274^*^	0.13885^*^	0.30822^**^	−0.09126	0.12728^*^	0.29584^**^	0.11109	0.12724
NP						0.31088^**^	0.2133^*^	−0.1037	0.05901	−0.21643^*^	−0.42423^**^	0.05928
PL							0.54372^**^	−0.24504^**^	0.11983^*^	−0.14073^*^	−0.40921^**^	0.1172
TNG								−0.04898	0.22772^*^	0.09622	−0.24709^**^	0.22528^*^
GW									0.21795	0.39876^**^	0.11883	0.21965^**^
TGW										0.15764^**^	0.06094	0.99881^**^
SL											0.33344^**^	0.15588^*^
SLWR												0.06269

Note: ^*^Significant at 0.05 probability level, ^**^highly significant at 0.01 probability level, PH: plant height; FLR: flag leaf length to width ratio; NT: number of tillers per hill; DF: days to flowering; DM: days to maturity; LA: leaf area; NP/H: number of panicles per hill; PL: panicle length; TNG/P: total number of grain per panicle; 100 GW: one hundred grain weight; TGW/H: total grain weight per hill; SL: seed length; SLWR: seed length width ratio; Y/ha: yield in t/ha.

**Table 6:** Grouping of 45 genotypes according to cluster analysis.

Cluster number	Number of genotypes	Genotypes
I	35	GN1, GN2, GN3, GN4, GN5, GN6, GN7, GN8, GN9, GN10, GN11, GN12, GN13, GN14, GN15, GN16, GN17, GN18, GN19, GN20, GN21, GN22, GN23, GN24, GN25, GN26, GN27, GN28, GN29, GN30, GN31, GN32, GN33, GN34, GN35
II	2	G36, G38
III	3	G37, G40, G41
IV	4	G39, G42, G44, G45
V	1	G43

Note: GN1 = ML-1, GN2 = ML-2, GN3 = ML-3, GN4 = ML-4, GN5 = ML-5, GN6 = ML-6, GN7 = ML-7, GN8 = ML-8, GN9 = ML-9, GN10 = ML-10, GN11 = ML-11, GN12 = ML-12, GN13 = ML-13, GN14 = ML-14, GN15 = ML-15, GN16 = ML-16, GN17 = ML-17, GN18 = ML-18, GN19 = ML-19, GN20 = ML-20, GN21 = ML-21, GN22 = ML-22, GN23 = ML-23, GN24 = ML-24, GN25 = ML-25, GN26 = ML-26, GN27 = ML-27, GN28 = ML-28, GN29 = ML-29, GN30 = ML-30, GN31 = ML-31, GN32 = MR 219, GN33 = MR 220, GN34 = MR 253, GN35 = MR 264, GN36 = Binadhan-4, GN37 = Binadhan-5, GN38 = Binadhan-6, GN39 = Binadhan-7, GN40 = Binadhan-10, GN41 = Binadhan-8, GN42 = IRATOM-38, GN43 = Binasail, GN44 = VN-124, and GN45 = VN-121.
